# ClinicNet: machine learning for personalized clinical order set recommendations

**DOI:** 10.1093/jamiaopen/ooaa021

**Published:** 2020-06-28

**Authors:** Jonathan X Wang, Delaney K Sullivan, Alex C Wells, Jonathan H Chen

**Affiliations:** Department of Medicine, Stanford University School of Medicine, Stanford, California, USA

**Keywords:** clinical decision support systems, precision medicine, electronic health records, order sets, deep learning

## Abstract

**Objective:**

This study assesses whether neural networks trained on electronic health record (EHR) data can anticipate what individual clinical orders and existing institutional order set templates clinicians will use more accurately than existing decision support tools.

**Materials and Methods:**

We process 57 624 patients worth of clinical event EHR data from 2008 to 2014. We train a feed-forward neural network (ClinicNet) and logistic regression applied to the traditional problem structure of predicting individual clinical items as well as our proposed workflow of predicting existing institutional order set template usage.

**Results:**

ClinicNet predicts individual clinical orders (precision = 0.32, recall = 0.47) better than existing institutional order sets (precision = 0.15, recall = 0.46). The ClinicNet model predicts clinician usage of existing institutional order sets (avg. precision = 0.31) with higher average precision than a baseline of order set usage frequencies (avg. precision = 0.20) or a logistic regression model (avg. precision = 0.12).

**Discussion:**

Machine learning methods can predict clinical decision-making patterns with greater accuracy and less manual effort than existing static order set templates. This can streamline existing clinical workflows, but may not fit if historical clinical ordering practices are incorrect. For this reason, manually authored content such as order set templates remain valuable for the purposeful design of care pathways. ClinicNet’s capability of predicting such personalized order set templates illustrates the potential of combining both top-down and bottom-up approaches to delivering clinical decision support content.

**Conclusion:**

ClinicNet illustrates the capability for machine learning methods applied to the EHR to anticipate both individual clinical orders and existing order set templates, which has the potential to improve upon current standards of practice in clinical order entry.

## LAY SUMMARY 

Scientific advances have led to a wealth of advances in medicine, but the escalating complexity makes it difficult for clinicians to learn how to efficiently use all patient information and optimize practice to the highest quality possible. In this study we develop ClinicNet, a recommender algorithm that anticipates clinical items (medications, procedures, consults, etc.) a clinician will order in the hospital based on prior similar cases. This is similar to online recommender systems that automatically anticipate your interests and needs. With ClinicNet, we can automatically generate lists of clinical order suggestions with greater accuracy than both conventional algorithmic approaches and checklists manually produced by human committees. We further develop an algorithm application to automatically guide clinicians toward existing decision support tools currently available but often overlooked in hospital systems.

## INTRODUCTION

Modern medicine is marked by undesirable clinical practice variability due both to the intractability of manually assimilating vast bodies of medical information and consistently applying such knowledge at scale. High quality, up-to-date, and effective clinical decisions require a physician to understand a large and growing amount of medical information.^1^ Expert knowledge is potent, but the difficulty of maintaining and reproducing such expertise means that it is essentially impossible to deliver it consistently and at scale without support systems.^2^ Without support systems and alternative information sources, physicians will be left to rely on personal intuition in the face of ever-escalating complexity of medical information.^3–5^

The United States has seen the widespread adoption of electronic health records (EHRs) in over 80% of hospitals, especially after recent reforms such as the HiTech act (2009) and Medicare Access and CHIP Reauthorization Act of 2015.^6^^,^^7^ EHRs support new tools such as computerized physician order entry (CPOE) that reduce medication errors, increase efficiency, and save hospitals money over the previous alternative of handwriting orders on paper.^8–11^ Though EHRs have provided many added benefits to patient care,^12^^,^^13^ the increased screen time from using EHR and CPOEs appears to be highly correlated with physician stress and burnout.^14–16^ This may lead to lower-quality care for patients.^17^^,^^18^ The development of clinical decision support systems that provide physicians with computerized assistance in clinical decisions^19^ are promising as they may help reduce screen time and prevent burnout among physicians, while improving the consistency and quality of care in the clinic.^20^^,^^21^

One common form of clinical order decision support used in clinical practice is institutional order sets. For a given patient, clinicians can search for and select from a pre-defined order set that may help inform what clinical items to order for different clinical scenarios (eg, blood transfusion process, admission for pneumonia, routine post-surgical care). These preformed templates consist of lab tests, medications, procedures, and other orders as determined by clinical committees.^22^ As clinical knowledge advances, order sets must be manually updated to stay current with medical guidelines and the availability of new orders, a process that is often inefficient and delivers unsatisfactory results.^23^^,^^24^

The availability of electronic medical data has laid the foundation for algorithmic approaches. In existing vendor-based CPOE workflows, clinicians search for clinical orders and order sets by name to retrieve a list of options to select from. Algorithmically generated recommendations may work within that same workflow, but instead of awaiting the user’s manual input of search criteria, the recommended orders and order sets can already be presented as options based on the available patient-specific data, while still allowing users to ignore those suggestions and proceed with their usual manual search workflow.

Previous literature has demonstrated the efficacy of statistical models, such as latent Dirichlet allocation probabilistic topic models and machine learning models, to generate order recommendations analogous to Netflix or Amazon.com’s product recommender.^25–28^ These methods are not only more accurate than current standard of care clinical order set decision support templates, but also they are more scalable and personalized than manually developing thousands of custom order sets.^29^ These data-driven order sets demonstrate utility in potentially reducing length of stay as well as reducing cognitive workload.^28^^,^^30^^,^^31^ Deep neural networks in particular have made progress in speech recognition, object detection, financial forecasting, and a variety of other domains.^32–35^ In medicine, in particular, these models perform well on tasks such as readmission, length of stay, triage, diagnosis through image segmentation, and death.^36–41^ While deep learning algorithms have been investigated to support clinical decisions, further work is needed to understand their potential clinical applications.^42^^,^^43^ Neural network algorithms may capture complex non-linear relationships in the clinical order set prediction task that are not as well captured in classical recommender approaches such as association rules and matrix factorization.

Recent clinical user acceptability assessments express anxiety with the use of clinical order recommendations generated from algorithms especially due to difficult interpretability of many approaches.^44^ In addition to predicting clinical items, our study proposes an additional clinical application of recommending existing order sets to users. This problem structure combines both top-down and bottom-up approaches for the purpose of knowledge summarization and dissemination. It has additional clinical workflow advantages as clinicians are already more trusting and familiar with these order set templates authored by institutional committees rather than algorithms.^44^

We analyzed data from STAnford medicine Research data Repository (STARR) which includes 57 624 patients from 2008 to 2014. Anytime a clinician orders an order set or a clinical item from the EHR, we consider that an opportunity to anticipate or provide a personalized recommendation within the clinician workflow. We trained 2 artificial neural network models on a feature matrix consisting of 35 million clinical item entries (across 57 624 patients) and 19 661 features to predict order set template usage as well as individual clinical items, making use of information only provided prior to the time of prediction. Anticipating ordering behavior for both order sets and individual clinical items provide a personalized, data-driven, and automated way to potentially improve patient outcomes in comparison to existing institutional order sets.^45^

**Figure 1.**
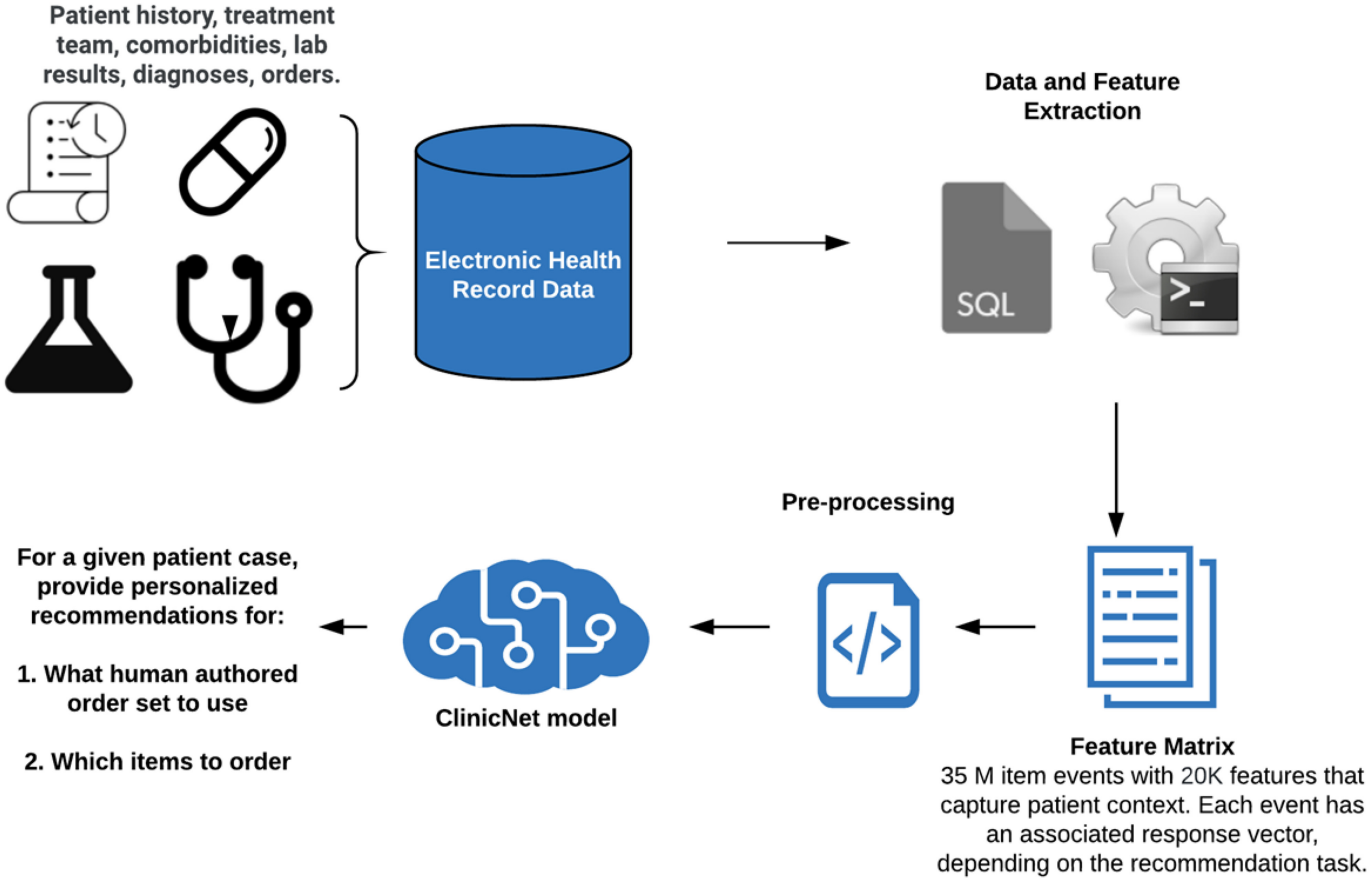
Schematic illustrating the prediction task. Electronic health record data from 57 624 patients are processed into a 35M by 20K feature matrix. Using this feature matrix, 2 different response vectors are created for order set usage and individual clinical items 24 hours after every instance an item is ordered. We then train and evaluate neural networks to predict order set usage and individual clinical items.

In this study, we aimed to determine whether machine learning methods trained on EHR data can predict individual clinical order decisions as well as the usage of order set templates more accurately than existing clinical decision support tools (Figure 1).

## MATERIALS AND METHODS

### Data source

De-identified Stanford Health Care (SHC) inpatient data from 2008 to 2014 was extracted through STARR.^46^^,^^47^ We define elements of the data repository as follows:

- Clinical item: Something that is associated with a patient in the EHR. Includes medications ordered, lab tests that resulted, ICD9 diagnosis codes, treatment teams, demographics, etc.
- Clinical order: A type of clinical item that a physician can order for a patient.
- Clinical item entry (event): A new record (row) that is generated when a clinical item is recorded in the EHR for a patient.
- Order set: Pre-defined templates of clinical orders.
- Patient record: Timestamped sequence of clinical item entries.

The data repository reflects the >74 000 patients hospitalized at SHC during the study period, including records of >55 million clinical item entries (events) drawn from >45 000 distinct clinical items (event types). Each patient is represented by several rows of data, where each of these rows corresponds to a single clinical item entry. Our study processed a random sample of 57 624 patients (35 million clinical item entries). The demographic makeup of the patients is depicted in Supplementary Figure S1.

### Data pre-processing and feature extraction

Clinical items were selected as follows. For medication clinical items, medications were grouped according to RxNorm mappings down to combinations of active ingredients and route.^46^ For example, clinical orders for both Norco and Vicodin pills were represented as “Acetaminophen-Hydrocodone (Oral)” while injections of metoprolol were represented as “Metoprolol (Intravenous)” regardless of dose or frequency. Information about a patient, such as race or sex, were represented as one-hot encoded features. The ICD9 coding hierarchy was rebuilt up to 3 digits (eg, 786·05 would count as 3 clinical items: 786·05, 786·0, and 786). Of the >45 000 resulting clinical items, we were only interested in clinical orders for the response vectors. After excluding non-order clinical items (eg, diagnosis codes, lab results) and “Nursing orders” that mostly reflect components of standard process templates like “Check vital signs” that would not be of interest for a prediction or recommender model, 14 914 clinical orders could be considered. In order to decrease the sparsity of our dataset we invoked the 80/20 power-law distribution^48^^,^^49^ of clinical orders to only include clinical orders occurring at least 256 times in the dataset, leaving 1639 clinical orders to consider, while still representing >90% of the clinical order events. For the order sets, a total of 610 order sets existed for the patients in our dataset and for each time that an order set was used, the order set identifier, the date the order set was used, and the patient on which the order set was used were recorded. For the features that comprised the feature matrix, the aforementioned 1639 clinical orders were used in addition to diagnosis codes, lab results, and treatment teams, as well as time features (eg, month and hour of the clinical item entry, which were sine- and cosine-transformed to represent the cyclical nature of these features), resulting in 6231 clinical items for the feature matrix. The features and response vectors are described in Supplementary Table S1.

### Construction of feature matrix and response vectors

Each row of data represented a clinical item entry (event) that was entered for a particular patient (eg, medication ordered, lab test resulted). Patients who had more entries recorded in the hospital comprised more rows of data than patients who had fewer entries. Features consisted of clinical items binned 4 time points: within 1 day prior, within 7 days prior, within 30 days prior, and any time prior. From the organization of the data described, each row in the feature matrix contained information about a patient’s record up to the point that the row was generated. Consider the following scenario for a new patient:

1. Patient receives an order of acetaminophen. The newly added row contains all zeroes because patient has no history at this point.
2. Patient receives another acetaminophen an hour later. This row has the number “1” for 4 acetaminophen features: ordered within 1 day prior (pre-1), 7 days prior (pre-7), 30 days prior (pre-30), and any time prior (pre-any). These “1’s” reflect the first order of acetaminophen.
3. Patient receives a third acetaminophen 10 days later. This row is “0” for acetaminophen pre-1 and pre-7 and “2” for acetaminophen pre-30 and pre-any, reflecting the 2 prior acetaminophens.
4. Patient receives aspirin a few minutes later. This row is identical to the previous row.
5. Patient receives a second order of aspirin several minutes later. In this row, all 4 aspirin features are “1” (in previous rows, the aspirin features were “0”). The acetaminophen features, again, would remain the same as in the previous 2 rows.

Each response variable was a binary variable representing whether a physician ordered that clinical item or used that order set within the next day (post-1). In the example scenario, row #3 would be “1” for acetaminophen post-1 (note: the current order of acetaminophen is included) and “1” for aspirin post-1 but all remaining response variables would be “0”.

### Training, validation, and test sets

The data were partitioned into training, validation, and test sets by a 70/15/15 split such that no patient appeared in multiple datasets. In order to prevent temporal leakage, the data splits were then subsetted such that the training set only contained entries from before the year 2011, the validation set only contained entries from 2011, and the test set only contained entries after 2011. For the order set prediction task, to mitigate data sparsity, only entries that had at least 1 order set used within the next 24 hours were retained. The partitioning of the data is detailed in Supplementary Figure S2. All count data were log2-transformed and all data were *z*-score standardized.

### Baselines

Our technical baseline model consisted of a logistic regression model trained with a binary cross-entropy loss function and a vanilla stochastic gradient descent optimizer with a 0.01 learning rate. For both the clinical item and order set prediction tasks, the model was trained for 1 epoch on the entire dataset.

The study additionally implemented 2 baselines that serve as heuristics for existing clinical decision support standard of care. For individual orders, we compared our performance to the usage of existing institutional order sets. For this comparison, we subsetted our test set to contain any instance where an item is ordered from within an order set. Our baseline was created using the full contents of the order set as a prediction. When a single clinical item was ordered from 2 order sets, we excluded this from our comparison (which only occurred for 0.8% of all orders).

For the order set prediction task, limited benchmarks exist in standard clinical practice, thus we developed a baseline using readily available EHR filters by admissions frequencies. We queried across the entire 55 million rows of the dataset to count the number of times an order set was ordered within 2 days for a given admission diagnosis that was collapsed to 3 digits (eg, ICD9 008, ICD9 009, ICD9 010). We used these counts to generate prediction probabilities scaled to a range from 0 to 1. If a given admission diagnosis had insufficient data with fewer than 50 total order set usages, then the “bestseller” list of most common order sets was used as a replacement (106 diagnoses were dropped out of 748 diagnoses and 18 930 order set usages were dropped out of 3 358 903 order set usages). If a patient item in the test set did not have an associated admission from our query, we replaced this with the bestseller list as well (194 893 rows did not have a corresponding admission out of 3 657 826 rows in the test set prior to filtering for temporal leakage).

### ClinicNet architecture

We developed all models using TensorFlow 2.0a and Python 3.6.^48^^,^^50^ ClinicNet architecture consists of a feed-forward neural network. Feed-forward networks have advantages over traditional machine learning approaches as they achieve full generality as well as the universal approximation property.^51–55^ We performed a hyperparameter search (Supplementary Table S2) on a random set of 50 000 rows of training data. These hyperparameters included batch normalization,^56^ number of hidden units, number of hidden layers, dropout,^57^ weight value in loss function, and L2 regularization. The ClinicNet models were all trained using Nesterov Adam optimizer for 1 epoch. While Adam is RMSprop with momentum,^58^ Nesterov Adam is RMSprop with Nesterov momentum, which is an often empirically superior form of momentum.^59^ This optimizer appears to achieve quicker, more stable learning for most tasks compared to Adam. We found that binary cross-entropy weighted by a constant true value which served as a hyperparameter (Equation 1) ultimately yielded the best results

(1) $$\left[-\frac{1}{m}\sum_{i}^{m}wy^{\left(i\right)}\log\left({\hat{y}}^{\left(i\right)}\right)+\left(1-{\hat{y}}^{\left(i\right)}\right)l\mathrm{og}\left(1-\;{\hat{y}}^{\left(i\right)}\right)\right]+\left[\frac{\lambda }{2}\sum_{l}^{L}{w_{l}}^{2}\right].$$

In the cross-entropy loss weighted by true value function, *m* is number of training examples, *w* is the positive weight, *y*^(^^*i*^^)^ is the true label for a given clinical item post-24 hours, *ŷ*^(^^*i*^^)^ is the predicted label for a given clinical item post-24 hours, λ is the L2 regularization weight, *L* is the total number of layers in the neural network, and *w_l_* are the weights of layer l in the model.

### Evaluation

To summarize performance across a range of thresholds, we used average precision^60^ and the area under the receiver operating characteristics (AUROC).^61^ For the institutional order set baseline comparison used in the individual clinical order prediction task, we thresholded ClinicNet and logistic regression models to binary values that achieve similar performance on recall to fairly compare F1 and precision to this baseline. For all reported metrics, we calculated patient-level confidence intervals (CIs) and average scores (reported values) through bootstrapping 10 000 rows per sample and 1000 iterations from the test set. Bootstrap sampling was randomized by patient so we were able to get patient-level evaluation metrics such that patients with more clinical item entries did not carry more weight in evaluation. Graphs were generated using patient-level statistics as well. CIs here apply to our model’s performance on the test set at the patient level, but because the rows in our test set are likely correlated, they may be an overestimate of confidence.

### Ethics

All research performed and methods described herein were approved by the Stanford University School of Medicine and the research compliance office’s Institutional Review Board panel at Stanford University.

## RESULTS

### Characteristics of feature matrix and response vectors

**Figure 2.**
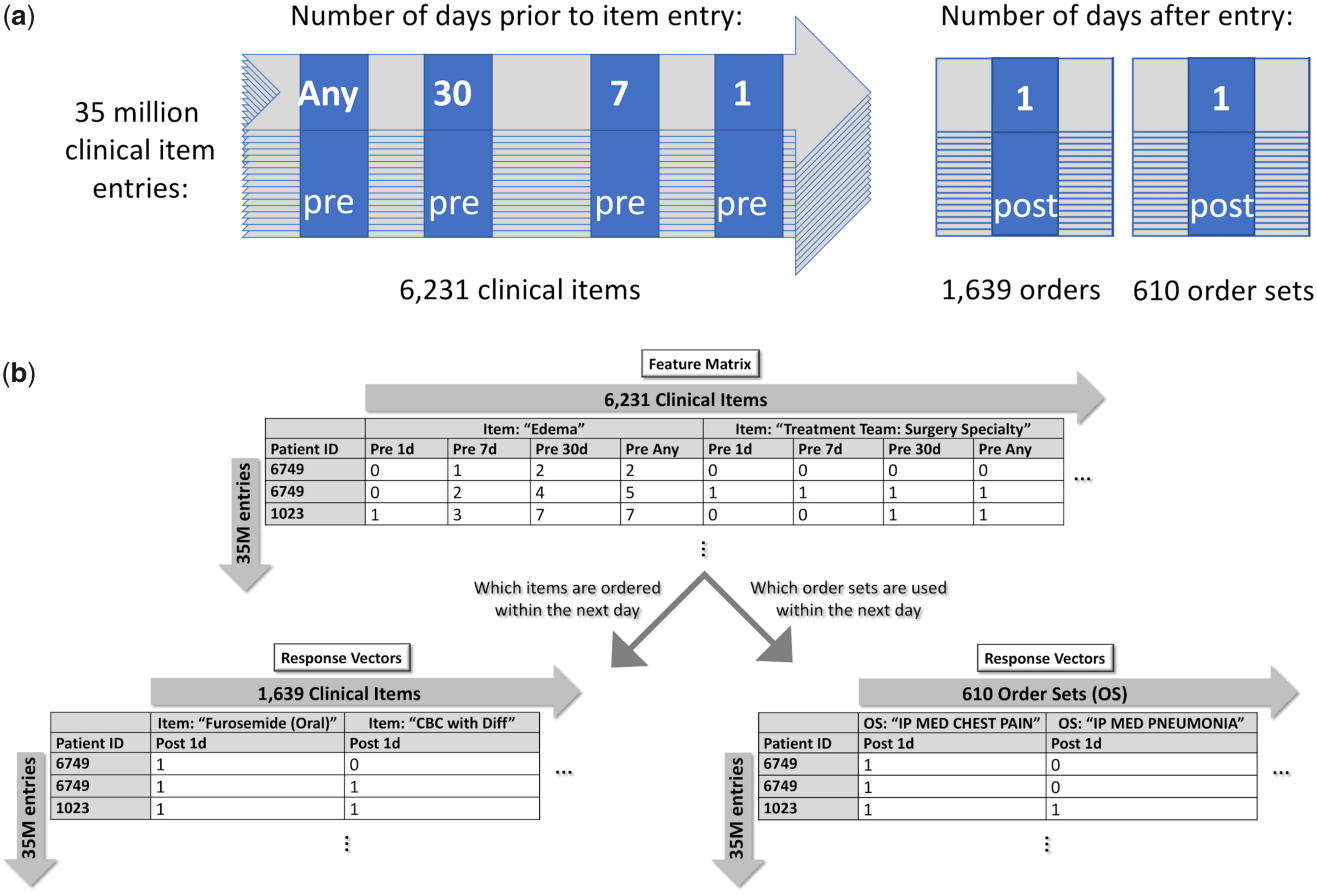
Organization of clinical data. (A) Modeling of patients as a timeline of clinical item entries. Each time a clinical item is entered for the patient makes up a new entry. For each entry, items associated with that patient within 4 time points prior to that entry (pre) are used as features. Orderable items (ie, items that a physician could order) and human-authored order sets associated with that patient within 1 day after that entry (post) are used as response variables. (B) Example of what the feature matrix and response vectors might look like. Here, we see that, at 1 time point, a diagnosis of edema had been made twice in the past (pre-any) for patient 6749 and, at another (later) time, edema had been entered 5 times in the past for that patient. In both cases, the patient was ordered furosemide and the order set, “IP MED CHEST PAIN,” was also used within the next day.

From the EHR of a tertiary academic hospital, we randomly sampled 57 624 patients represented by 35 million rows of clinical item event data. Each patient in the EHR was represented as a timeline of clinical items (Figure 2A), therefore each patient made up several rows of data.

For features, we curated 6231 clinical items (consisting of clinical orders placed, demographics, ICD9 diagnoses codes, lab results, treatment teams, etc.) and binned them at 4 time points: within 1 day prior, within 7 days prior, within 30 days prior, and any time prior (Supplementary Table S1). Following feature selection, whereupon over 5000 low-variance (standard deviation < 0.01 in training set) features were removed, 19 661 features resulted, which were represented by the columns of the feature matrix (Figure 2B).

We constructed 2 sets of response vectors, one for the task of predicting individual orders (1639 orders total) and another for the task of predicting order set usage (610 order sets total) (Figure 2B).

### Algorithm performance

**Figure 3.**
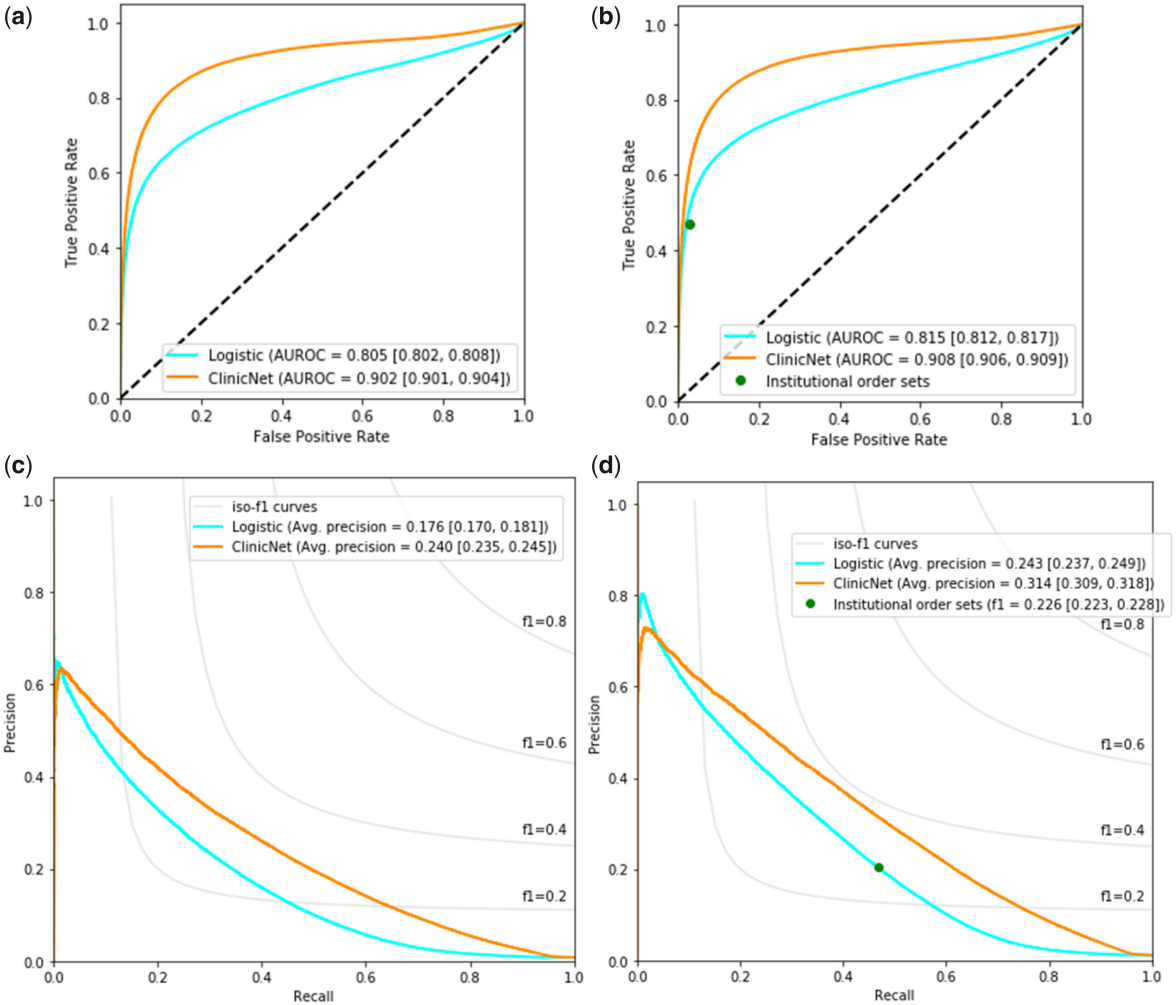
The classification performance on predicting individual clinical items according to the overall test set and to instances when institutional order sets were used. (A) ROC curve for overall test set. (B) ROC curve for item instances where institutional order sets were used. (C) Precision–recall curve for overall test set. (D) Precision–recall curve for item instances where institutional order sets were used. The performance was measured based on AUROC and average precision, which were bootstrapped with a sample size of 10 000 for 1000 iterations to obtain 95% confidence intervals located in brackets. Evaluation was performed at the patient-level rather than the clinical item-level. *Abbreviation:* AUROC: area under the receiver operating characteristics.

Figure 3 shows the performance for the individual clinical item prediction task on the test set. When evaluating against the overall test set, logistic regression had a 0.805 (95% CI 0.802–0.808) AUROC and 0.176 (95% CI 0.170–0.181) average precision while ClinicNet performed better with a 0.902 (95% CI 0.901–0.904) AUROC and 0.240 (95% CI 0.235–0.245) average precision. The performance was also evaluated against a subset of the test set which consisted of instances when a physician pulled up an institutional order set to order an item for further comparison. ClinicNet significantly outperformed in AUROC (0.908 with 95% CI from 0.906 to 0.909) and average precision (0.314 with 95% CI from 0.309 to 0.318) when compared to a logistic regression model and using institutional order sets as a set of predictions.

Table 1 presents the performance of the model on the institutional order set subset task when thresholded to similar levels of recall. ClinicNet had significantly higher F1 scores (0.378 with 95% CI 0.375–0.381) than both the institutional order set baseline (0.226 with 95% CI 0.223–0.228) and logistic regression (0.285 with 95% CI 0.280–0.289).

**Table 1.** Precision, recall, and F1 score of ClinicNet, institutional order sets, and logistic regression when thresholded to similar levels of recall

	Evaluation metrics			
Models	Precision (95% CI)	Recall (95% CI)	F1 (95% CI)	AUROC (95% CI)
Logistic	0.204 (0.200–0.208)	0.469 (0.464–0.473)	0.285 (0.280–0.289)	0.815 (0.812–0.817)
Institutional	0.149 (0.147–0.151)	0.463 (0.458–0.469)	0.226 (0.223–0.228)	
ClinicNet	**0.317 (0.314–0.320)**	0.468 (0.463–0.472)	**0.378 (0.375–0.381)**	**0.908 (0.906–0.909)**

*Note:* As institutional order sets consist of a single threshold point, AUROC is left blank. Metrics were bootstrapped with a sample size of 10 000 for 1000 iterations to get reported CIs. Evaluation was performed at the patient-level rather than the clinical item-level. The following thresholds were used: Logistic regression = 0.11, ClinicNet = 0.50. Bold indicates highest metric.

*Abbreviations:* AUROC: area under the receiver operating characteristics; CI: confidence interval.

**Figure 4.**
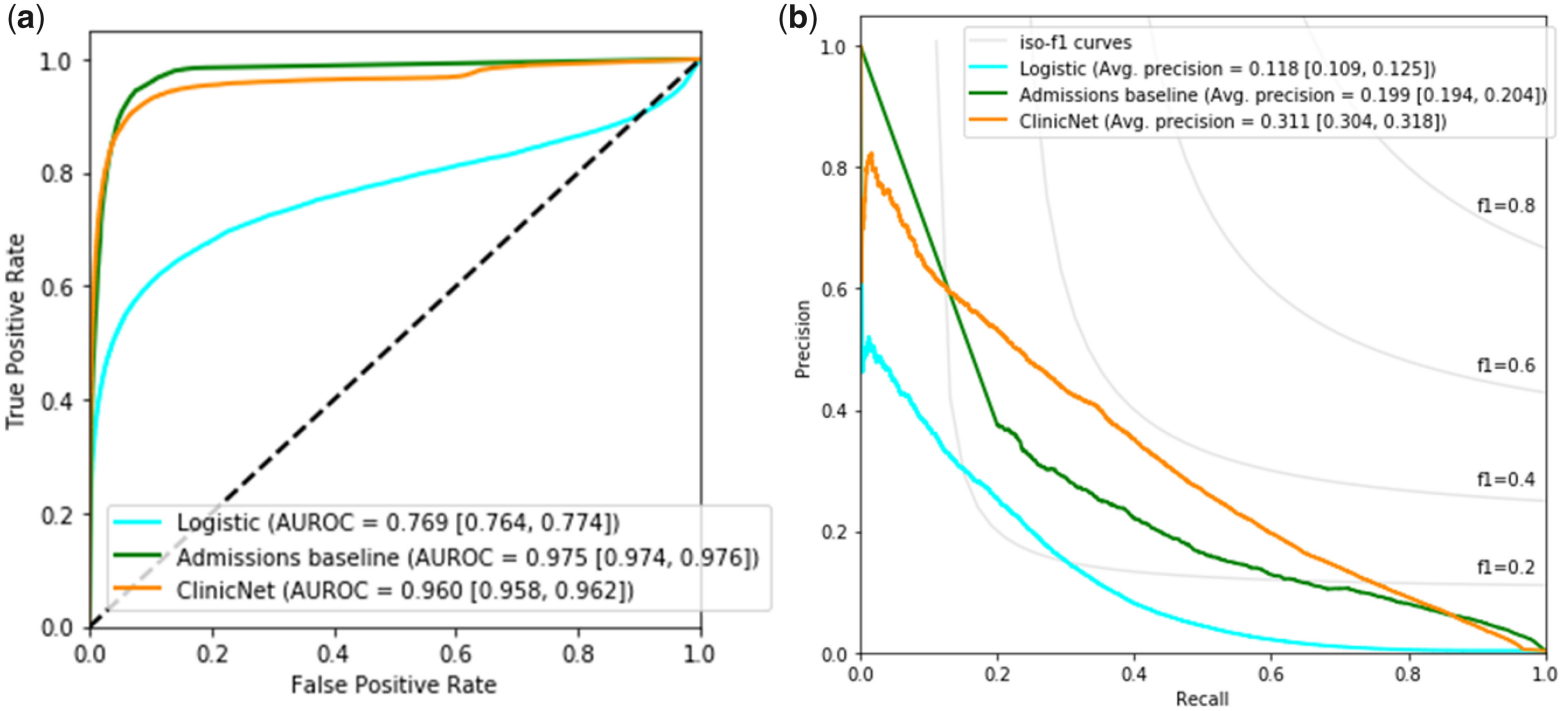
The classification performance on predicting usage of existing order set templates across ClinicNet, an admissions baseline, and logistic regression evaluated on the test set. (A) ROC curve. (B) Precision–recall curve. The performance was measured based on AUROC and average precision, which were bootstrapped with a sample size of 10 000 for 1000 iterations to obtain 95% confidence intervals located in brackets. Evaluation was performed at the patient-level rather than the clinical item-level. *Abbreviation:* AUROC: area under the receiver operating characteristics.

Prediction performance for usage of existing institutional order sets is presented in Figure 4. ClinicNet was compared to logistic regression and a baseline using the most frequent items associated with a given patient admission diagnosis across a range of thresholds. ClinicNet performed best in average precision (0.311 with 95% CI 0.304–0.318) compared to both logistic (0.118 with 95% CI 0.109–0.125) and admission baselines (0.199 with 95% CI 0.194–0.204). In AUROC, admissions baseline (0.975 with 95% CI 0.974–0.976) performs slightly better than ClinicNet (0.960 with 95% CI 0.958–0.962), with a greater difference in performance compared to logistic (0.769 with 95% CI 0.764–0.774). The admissions baseline and ClinicNet have 2 intersection points on their precision–recall curves, which suggest there are thresholds where one performs better than the other in precision and recall.

## DISCUSSION

In this study, we developed ClinicNet, a personalized clinical order decision recommender which leverages deep neural networks applied to 57 624 patients worth of EHR data from Stanford. We trained, developed, and tested 2 different feed-forward neural network models: (1) the traditional task of recommending custom order sets to physicians from 1639 orderable items and (2) a newer proposed workflow of recommending which order set to use out of currently available institutional order sets. Our new problem structure is of interest to us as initial assessment of algorithms used to predict custom order sets have been met with negative feedback out of anxiety over algorithms that are difficult to interpret.^44^ This problem structure leverages existing institutional order sets that clinicians trust and are familiar with. As the dataset contained over 35 million rows from 57 624 patients, we elected to use deep learning for our prediction tasks as deep learning models are suitable for learning complex patterns from large amounts of data.^62^ Our model performance was evaluated by comparing AUROC, F1, precision, and recall to both technical and standard of care benchmarks.

ClinicNet compares favorably to our prior work using episode mining recommender algorithms^29^ and probabilistic topic models,^25^ though differing problem structure limits direct comparison. ClinicNet outperformed baselines in all evaluation metrics for the clinical item prediction task, and only performed worse in AUROC for the order set task when compared to the admissions baseline. Interestingly, when we looked at the clinical item-level evaluation metrics (as opposed to the patient-level metrics used for the results of the study) we found that ClinicNet outperformed in both metrics (AUROC 0.967 vs AUROC 0.950). Additionally, ClinicNet outperforms admissions baseline in average precision, a more realistic evaluation metric for this task. AUROC is an important general measure, but clinicians may not be interested in how well sorted the list of 610 order set options is at the bottom. Recommender systems allow a user to narrow their focus to a top X most likely choices, requiring an attention threshold reflected better using average precision. Our study shows that deep neural networks may be a good choice of algorithm to be used to suggest order sets and order set usage to clinicians, as logistic regression did not surpass the standard of care baselines. It also demonstrates the potential for algorithms to anticipate order set usage as a form of clinical decision support.

The human-authored order set baseline, wherein individual items were recommended on the basis of order sets, exhibited a high recall (true positive rate). This makes sense because an order set has a high coverage of clinical items so even though there will be a significant number of false positives, there will be a high fraction of items from the order sets used on the patient that are part of the set of items that were actually ordered for the patient. However, at the same recall as the human-authored order set baseline, logistic regression and the ClinicNet model both show much higher precision. Highly precise order sets are of great clinical interest. Such an order set only contains items that are likely to be important in the management of an individual patient and exclude those that are not clinically useful. Conversely, order sets that demonstrate low precision contain extraneous items not indicated for a specific patient and, if ordered, may contribute to unnecessary testing and inflated healthcare costs.

Several limitations of this study exist. For one, the prediction tasks are predicated on the response variables, which is the actual decision that a physician ends up making for a patient, being the “gold standard.” Thus, the objective of the neural network is to learn to predict the decisions made by physicians. However, physicians can and do make mistakes, therefore this is not a true gold standard since the decisions that a physician makes may not necessarily be the ones that are the best for a patient.^29^ While a potential solution to this limitation includes expert vetting of the clinical decisions made by physicians and considering the clinical outcome of a patient given a set of clinical decisions made by a physician, this task is largely infeasible given the size of the dataset used in the training and evaluation of ClinicNet. Another limitation is that ClinicNet was trained on EHR data solely from SHC. As a result, this model might not necessarily generalize well to other patient populations or hospitals and may need to be retrained if used on other EHR data.

Finally, there are many barriers to enable this fully integrated vision with vendor-based CPOE. For instance, hospitals have different EHR systems and introducing new software has a number of administrative and technical protocols to be approved. However, simpler options including view-only suggestions are already viable through Fast Healthcare Interoperability Resources interfaces. Future work will need to further address these implementation complexities. This includes user interface testing for clinical usability and acceptability as well as exploring alternative algorithmic approaches such as standard machine learning methods and recurrent neural networks, though our preliminary attempts at such approaches have not yet performed as well as the approaches described here.

## CONCLUSIONS

In conclusion, ClinicNet, a deep neural network model, outperformed technical and standard of care benchmarks in terms of multiple metrics toward predicting clinical care decisions. Our work illustrates the possibility for an automated, scalable system to dynamically anticipate what clinical order and order sets a clinician needs through algorithmically inferring patient context in the EHR. The clinical insight provided may improve upon the consistency and quality of the current standard of care.

## DATA AVAILABILITY 

The clinical data originates from the Stanford University Hospital and can be accessed for research purposes via the Stanford Research Repository (STARR) (https://med.stanford.edu/starr-tools.html). The authors have made the code available on the HealthRex Github repository https://github.com/HealthRex/CDSS/. Data for results can be found on Dryad (doi:10.5061/dryad.msbcc2fvm). 

## FUNDING

This research was supported in part by the NIH Big Data 2 Knowledge initiative via the National Institute of Environmental Health Sciences under award number K01ES026837, the Gordon and Betty Moore Foundation through Grant GBMF8040, a Stanford Human-Centered Artificial Intelligence Seed Grant, and a Stanford Undergraduate Advising and Research Grant. This research used data or services provided by STARR (STAnford medicine Research data Repository), a clinical data warehouse containing live Epic data from Stanford Health Care (SHC), the University Healthcare Alliance (UHA), and Packard Children’s Health Alliance (PCHA) clinics and other auxiliary data from Hospital applications such as radiology PACS. The STARR platform is developed and operated by Stanford Medicine Research IT team and is made possible by Stanford School of Medicine Research Office. The content is solely the responsibility of the authors and does not necessarily represent the official views of the NIH or Stanford Healthcare.

## AUTHOR CONTRIBUTIONS

JXW and DKS had full access to all the data in the study and contributed equally to the work presented here. All authors provided contributions to generating models, baselines, and querying the dataset. All authors discussed content, revised the paper, and approved the final manuscript.

## SUPPLEMENTARY MATERIAL

Supplementary material is available at *Journal of the American Medical Informatics Association* online.

## Supplementary Material

ooaa021_Supplementary_Data
