# Supplementary material for: ClinicNet: machine learning for personalized clinical order set recommendations
Source: JAMIA Open. 2020 Jun 28;3(2):216–24. doi: 10.1093/jamiaopen/ooaa021 (PMC7382624; doi:10.1093/jamiaopen/ooaa021)
Supplement: ooaa021_Supplementary_Data [file ooaa021_supplementary_data.zip › ooaa021-Suppl_Data/Supplementary_Table_2.docx]

**Supplementary Table 2**

1. Order set prediction task

| **#** | **Neurons per layer** | **Learning rate** | **Loss function** | **weight** | **dropout** | **L2** | **batch-norm** | **Train F1** | **Val F1** |
| --- | --- | --- | --- | --- | --- | --- | --- | --- | --- |
| 1 | [1024] | 0.008 | binary | NA | 0.05 | 0.0025 | no | 0.08 | 0.05 |
| 2 | [2048, 1024] | 0.008 | weighted | 4 | 0.05 | 0.0025 | no | 0.16 | 0.24 |
| 3 | [2048, 1024] | 0.008 | weighted | heuristic | 0.05 | 0.0025 | no | 0.07 | 0.06 |
| 4 | [2048, 1024] | 0.008 | binary | NA | 0.05 | 0.0025 | no | 0.08 | 0.13 |
| 5 | [2048] | 0.008 | weighted | 4 | 0.05 | 0.0025 | no | 0.16 | 0.16 |
| 6 | [1024,512,256] | 0.008 | weighted | 4 | 0.05 | 0.0025 | no | 0.14 | 0.18 |
| 7 | [4096] | 0.008 | weighted | 4 | 0.05 | 0.0025 | no | 0.14 | 0.19 |
| 8 | [2048,1024] | 0.03 | weighted | 4 | 0.05 | 0.0025 | no | 0.10 | 0.11 |
| 9 | [2048,1024] | 0.001 | weighted | 4 | 0.05 | 0.0025 | no | 0.27 | 0.27 |
| 10 | [2048,1024] | 0.0003 | weighted | 4 | 0.05 | 0.0025 | no | 0.32 | 0.30 |
| 11 | [2048,1024] | 0.0001 | weighted | 4 | 0.05 | 0.0025 | no | 0.36 | 0.33 |
| 12 | [2048,1024] | 0.00003 | weighted | 4 | 0.05 | 0.0025 | no | 0.34 | 0.35 |
| 13 | [2048,1024] | 0.0001 | weighted | 3 | 0.05 | 0.0025 | no | 0.34 | 0.31 |
| 14 | [2048,1024] | 0.0001 | weighted | 5 | 0.05 | 0.0025 | no | 0.37 | 0.34 |
| 15 | [2048,1024] | 0.0001 | weighted | 6 | 0.05 | 0.0025 | no | 0.37 | 0.34 |
| 16 | [2048,1024] | 0.0001 | weighted | 5 | 0.1 | 0.01 | no | 0.33 | 0.31 |
| 17 | [2048,1024] | 0.0001 | weighted | 5 | 0.05 | 0.0025 | yes | 0.37 | 0.34 |
| 18 | [2048,1024] | 0.0001 | weighted | 5 | 0.0025 | 0.0008 | yes | 0.40 | 0.35 |
| 19 | [2048,1024] | 0.0001 | weighted | 5 | 0.0035 | 0.001 | yes | 0.40 | 0.35 |
| 20 | **[2048,1024]** | **0.0001** | **weighted** | **5** | **0.25** | **0.0025** | **yes** | **0.37** | **0.34** |

1. ClinicNet Clinical Item prediction Task

| **#** | **Neurons per layer** | **Learning rate** | **Loss function** | **weight** | **dropout** | **L2** | **batch-norm** | **Train F1** | **Val F1** |
| --- | --- | --- | --- | --- | --- | --- | --- | --- | --- |
| 1 | [2048,1024] | 0.0001 | weighted | 5 | 0.25 | 0.0025 | True | 0.29 | 0.29 |
| 2 | [2048,1024, 512] | 0.0001 | weighted | 5 | 0.25 | 0.0025 | True | 0.17 | 0.26 |
| 3 | [2048] | 0.0001 | weighted | 5 | 0.25 | 0.0025 | True | 0.29 | 0.29 |
| 4 | [4096] | 0.0001 | weighted | 5 | 0.25 | 0.0025 | True | 0.29 | 0.29 |
| 5 | [2048,1024] | 0.0003 | weighted | 5 | 0.25 | 0.0025 | True | 0.29 | 0.28 |
| 6 | [2048,1024] | 0.00003 | weighted | 5 | 0.25 | 0.0025 | True | 0.26 | 0.28 |
| 7 | [2048,1024] | 0.0001 | weighted | 7 | 0.25 | 0.0025 | True | 0.30 | 0.30 |
| 8 | [2048,1024] | 0.0001 | weighted | 8 | 0.25 | 0.0025 | True | 0.30 | 0.30 |
| 9 | [2048,1024] | 0.0001 | weighted | 6 | 0.25 | 0.0025 | True | 0.30 | 0.30 |
| 10 | [2048,1024] | 0.0001 | weighted | 7 | 0.15 | 0.0025 | True | 0.30 | 0.30 |
| 11 | [2048,1024] | 0.0001 | weighted | 7 | 0.25 | 0.001 | False | 0.32 | 0.31 |
| 12 | [2048,1024] | 0.0001 | weighted | 7 | 0.10 | 0.0003 | False | 0.34 | 0.32 |
| 13 | [2048,1024] | 0.0001 | weighted | 7 | 0.10 | 0.0003 | True | 0.34 | 0.32 |
| 14 | [2048,1024] | 0.0001 | weighted | 7 | 0.05 | 0.0001 | False | 0.35 | 0.33 |
| 15 | [2048,1024] | 0.0001 | weighted | 7 | 0.025 | 0.00003 | False | 0.35 | 0.32 |
| 16 | **[2048,1024]** | **0.0001** | **weighted** | **7** | **0.25** | **0.0001** | **False** | **0.35** | **0.33** |

**Supplementary Table 2**: List of hyperparameters used for ClinicNet models

*50,000 random rows of data

*heuristic used class (1-class frequency average)/class frequency average

*selected bold due to higher regularization with similar results, given that we will train even more data later
