# Supplementary material for: ClinicNet: machine learning for personalized clinical order set recommendations
Source: JAMIA Open. 2020 Jun 28;3(2):216–24. doi: 10.1093/jamiaopen/ooaa021 (PMC7382624; doi:10.1093/jamiaopen/ooaa021)
Supplement: ooaa021_Supplementary_Data [file ooaa021_supplementary_data.zip › ooaa021-Suppl_Data/Supplementary_Figure_1.docx]

**a d**
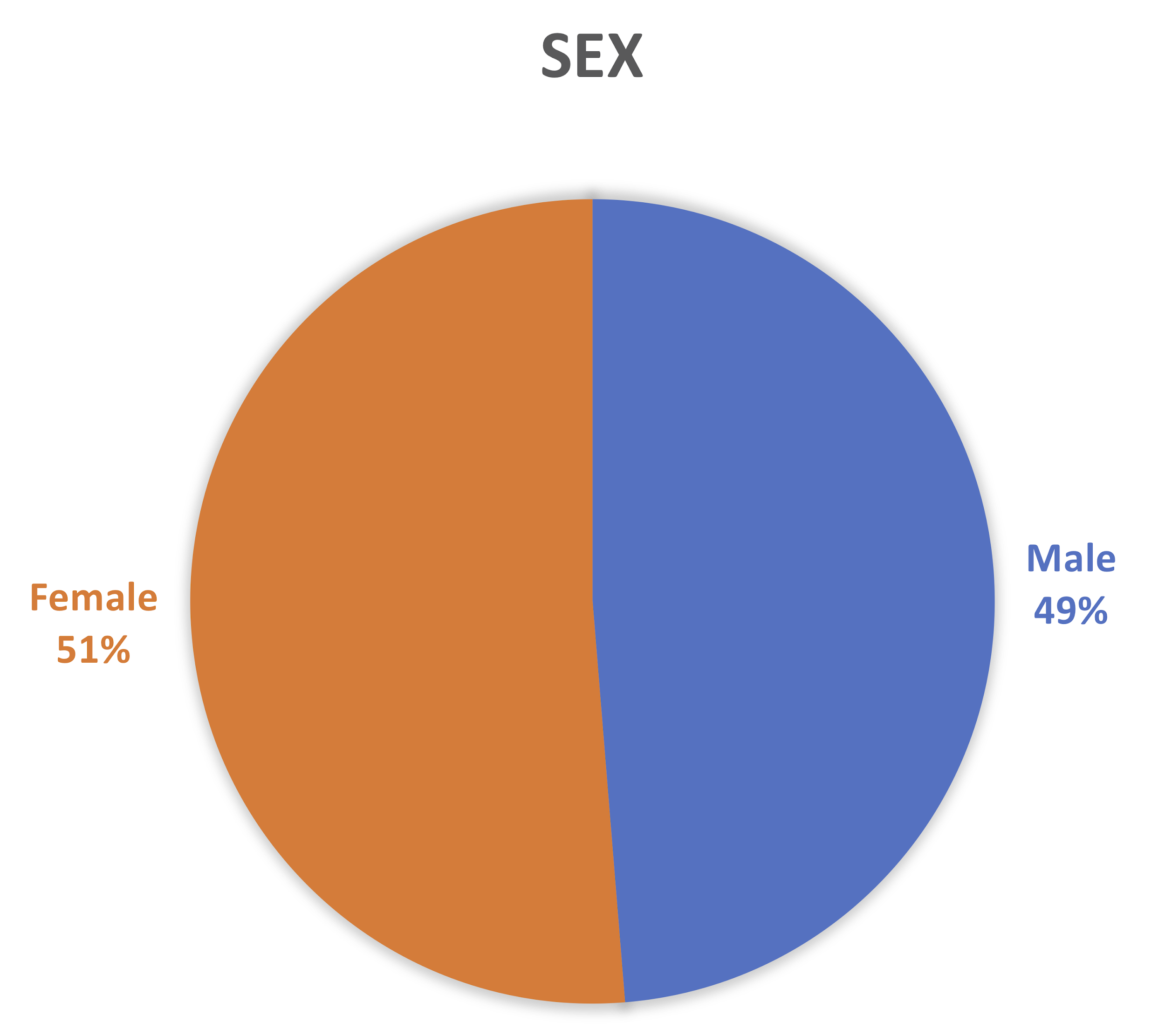

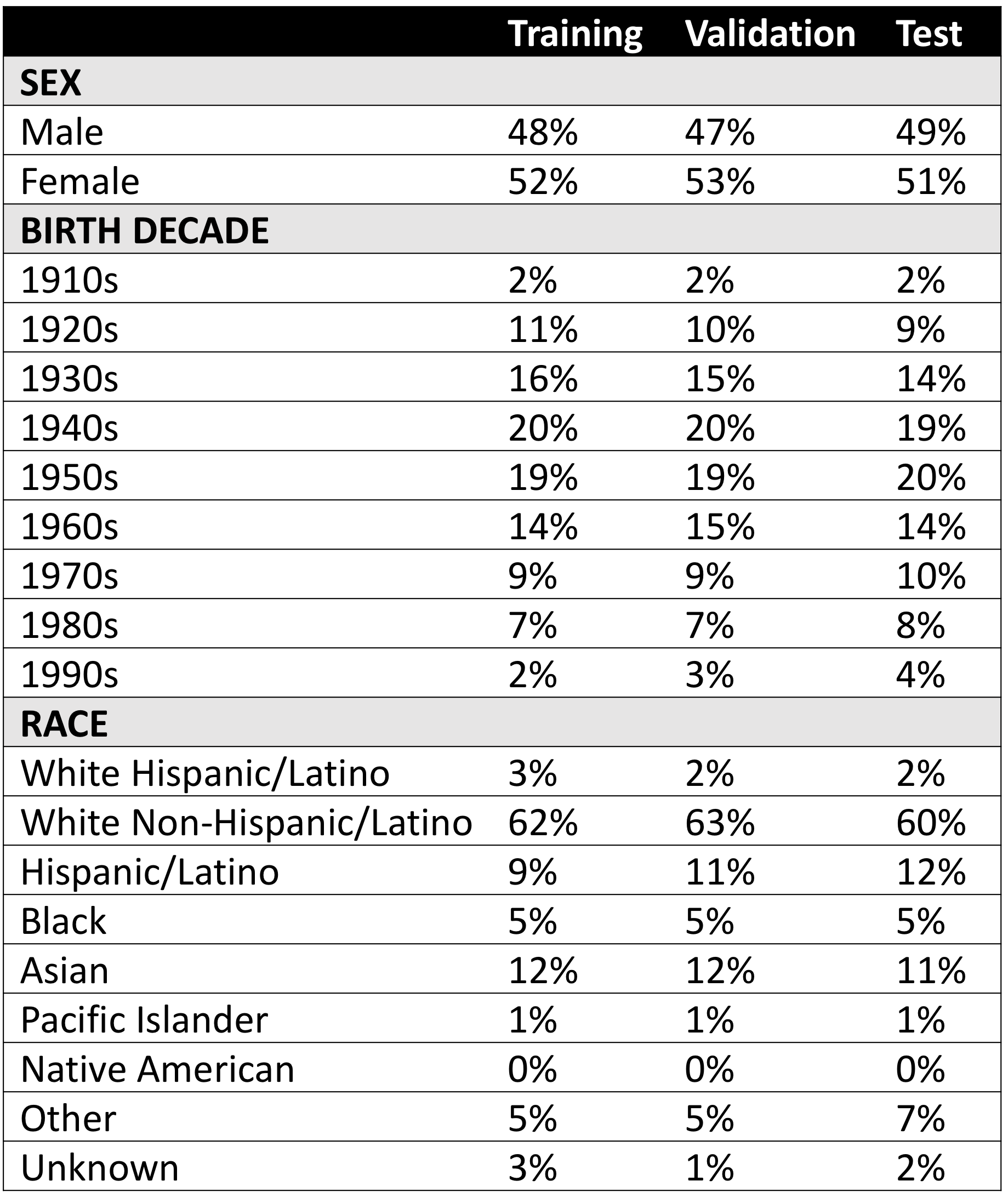


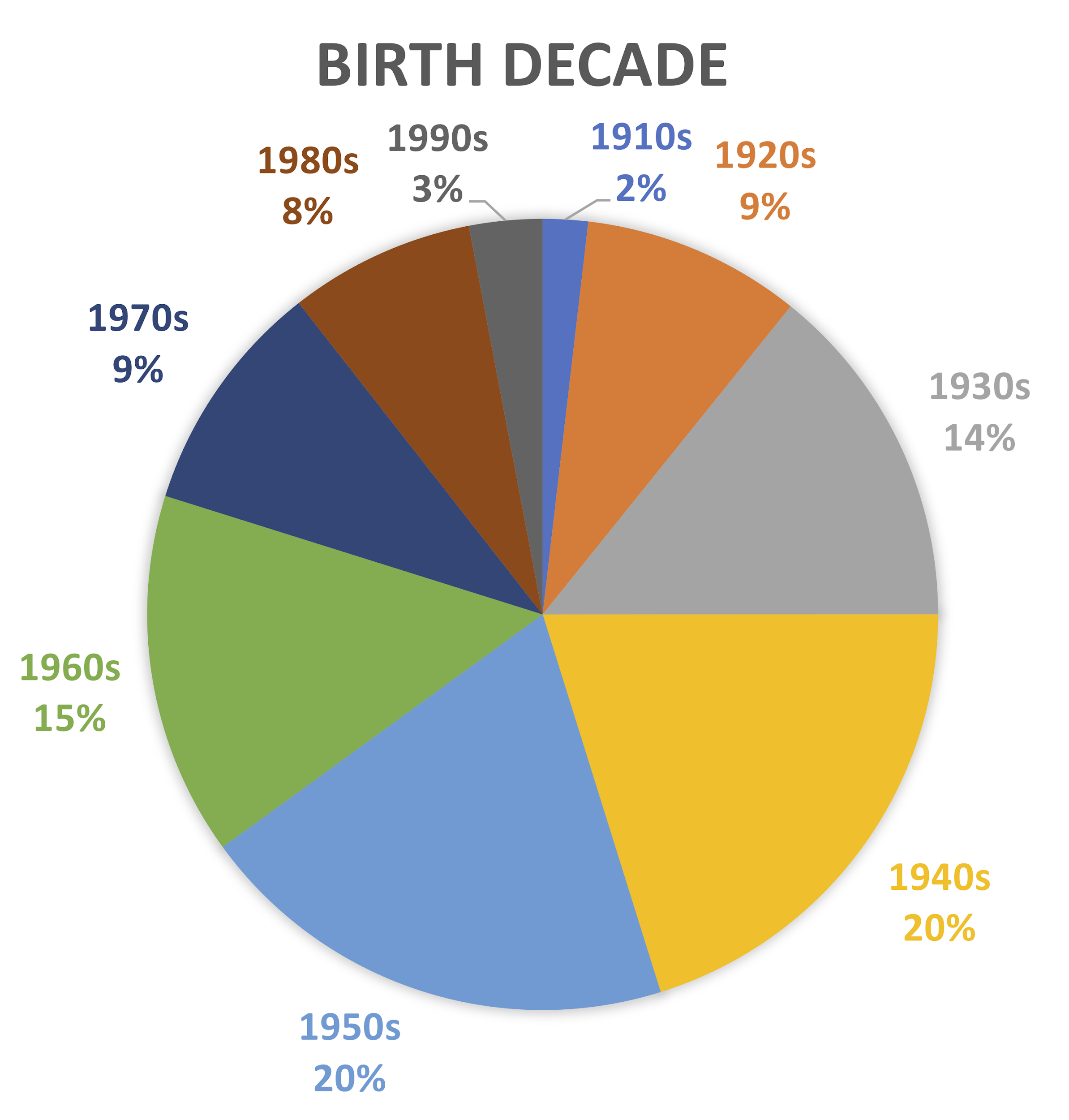


**b**


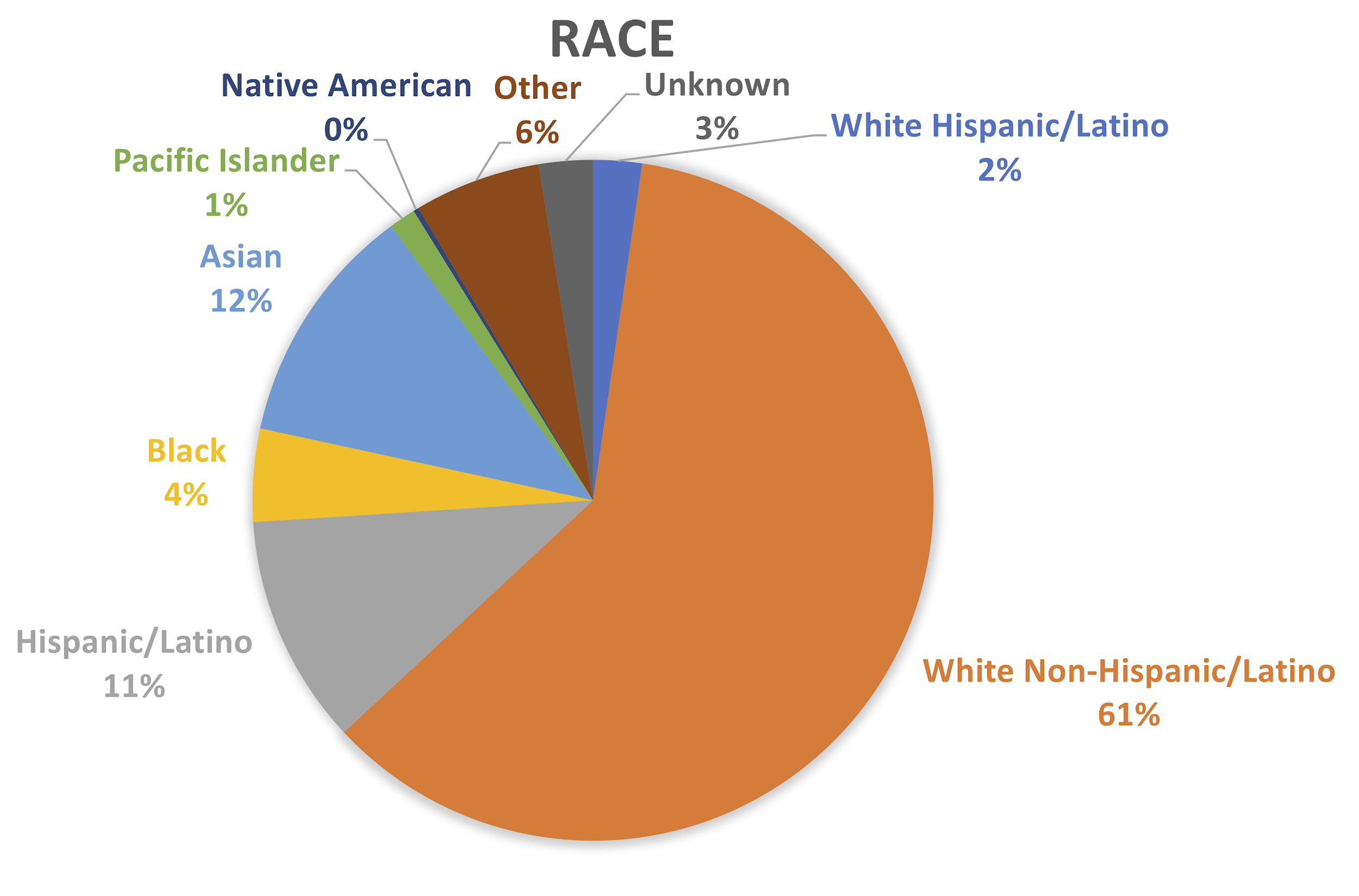


**c**

**Supplementary Figure 1**: Patient demographics

**a**, Distribution of sex. **b**, Distribution of birth decade. **c**, Distribution of race. **d**, Demographics for the training, validation, and test set split of the data.
