# Supplementary figures and images for: ClinicNet: machine learning for personalized clinical order set recommendations
Source: JAMIA Open. 2020 Jun 28;3(2):216–24. doi: 10.1093/jamiaopen/ooaa021 (PMC7382624; doi:10.1093/jamiaopen/ooaa021)

**
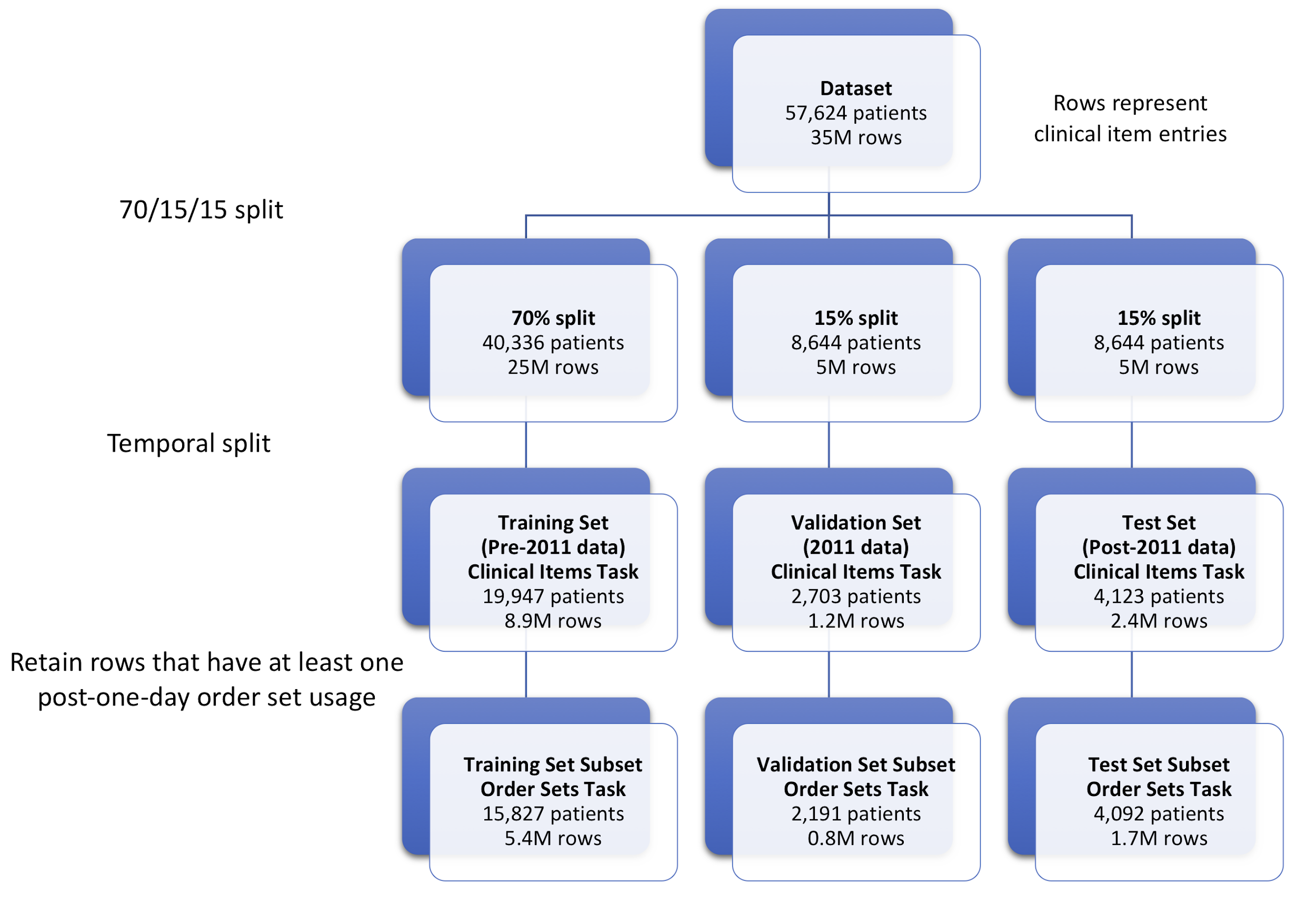
**

**Supplementary Figure 2**: Partitioning of the dataset

Supplement: ooaa021_Supplementary_Data [file ooaa021_supplementary_data.zip › ooaa021-Suppl_Data/Supplementary_Figure_2.docx]
